# A system to simultaneously detect tick-borne pathogens based on the variability of the 16S ribosomal genes

**DOI:** 10.1186/1756-3305-6-269

**Published:** 2013-09-18

**Authors:** Jana Melničáková, Marketa Derdáková, Imrich Barák

**Affiliations:** 1 Institute of Molecular Biology, Slovak Academy of Sciences, Dúbravska cesta 21, 845 51, Bratislava, Slovak Republic; 2 Institute of Zoology, Slovak Academy of Sciences, Dúbravska cesta 9, 845 06, Bratislava, Slovak Republic; 3 Institute of Parasitology, Slovak Academy of Sciences, Hlinkova 3, 040 01, Košice, Slovak Republic

**Keywords:** Tick-borne bacteria, DNA microarray, Quantitative PCR

## Abstract

**Background:**

DNA microarrays can be used to quickly and sensitively identify several different pathogens in one step. Our previously developed DNA microarray, based on the detection of variable regions in the 16S rDNA gene (*rrs*), which are specific for each selected bacterial genus, allowed the concurrent detection of *Borrelia* spp*., Anaplasma* spp*., Francisella* spp*., Rickettsia* spp*.* and *Coxiella* spp*.*

**Methods:**

In this study, we developed a comprehensive detection system consisting of a second generation DNA microarray and quantitative PCRs. New oligonucleotide capture probes specific for *Borrelia burgdorferi* s.l. genospecies and *Candidatus* Neoehrlichia mikurensis were included. This new DNA microarray system required substantial changes in solution composition, hybridization conditions and post-hybridization washes.

**Results:**

This second generation chip displayed high specificity and sensitivity. The specificity of the capture probes was tested by hybridizing the DNA microarrays with Cy5-labeled, PCR-generated amplicons encoding the *rrs* genes of both target and non-target bacteria. The detection limit was determined to be 10^3^ genome copies, which corresponds to 1–2 pg of DNA. A given sample was evaluated as positive if its mean fluorescence was at least 10% of the mean fluorescence of a positive control. Those samples with fluorescence close to the threshold were further analyzed using quantitative PCRs, developed to identify *Francisella* spp., *Rickettsia* spp. and *Coxiella* spp. Like the DNA microarray, the qPCRs were based on the genus specific variable regions of the *rrs* gene. No unspecific cross-reactions were detected. The detection limit for *Francisella* spp. was determined to be only 1 genome copy, for *Coxiella* spp. 10 copies, and for *Rickettsia* spp., 100 copies.

**Conclusions:**

Our detection system offers a rapid method for the comprehensive identification of tick-borne bacteria, which is applicable to clinical samples. It can also be used to identify both pathogenic and endosymbiontic bacteria in ticks for eco-epidemiological studies, tick laboratory colony testing, and many other applications.

## Background

Tick transmitted diseases are a serious and permanent public health problem. In Europe, the most frequent and most epidemiologically important vector is the hard tick *Ixodes ricinus*. It transmits viral, bacterial and protozoan agents to humans and animals. The most common and important tick-transmitted disease in the northern hemisphere, Lyme borreliosis, is caused by spirochetes from the *Borrelia burgdorferi* sensu lato (s.l.) complex. It currently includes 19 different genospecies [1]. The considerable genotypic and phenotypic heterogeneity of the *B. burgdorferi* s.l. complex has been linked to differences in pathogenicity, clinical symptoms and ecology [2-4]. The *Borrelia* genus also includes a second group of spirochetes, called the relapsing fever group. The spirochetes of this group are transmitted mainly by soft ticks, but can also utilize some hard ticks as vectors [5].

Anaplasmoses are also common tick-borne, zoonotic bacterial diseases. The causative agents are intracellular gram-negative bacteria that belong to the family Anaplasmataceae [6]. The genus *Anaplasma* consists of *Anaplasma marginale, Anaplasma ovis*, *Anaplasma bovis* and *Anaplasma platys.* While they are primary of veterinary significance, *A. phagocytophilum* can cause granulocytic anaplasmosis in humans as well as horses and dogs and tick-borne fever in ruminants [7]. A relatively new member of the family Anaplasmataceae is *Candidatus* Neoehrlichia mikurensis [8]. It infects endothelial cells and most infection symptoms depend on the physical status of the patient. The illness predominantly develops in immunocompromised patients [9-12].

Less common bacteria that may be transmitted by *Ixodes ricinus* amongst other tick species include *Francisella* spp*.* and *Coxiella* spp*. Coxiella burnetii* is the causative agent of Q-fever, which can be either an acute or chronic disease. *Francisella tularensis* causes tularemia, a febrile disease with myalgia and headache and when left untreated, it can cause a high mortality rate [13]. Most cases of disease caused by both *C. burnetii* and *F. tularensis* result from non-vector transmission.

Another European tick-borne obligate intracellular parasite, which is also globally distributed, is *Rickettsia* spp. The genus *Rickettsia* contains many species which form several biogroups, including the typhus fever group, the spotted fever group and the group causing tick-borne lymphadenopathy or *Dermacentor* spp. - borne necrosis - erythema - lymphadenopathy (TIBOLA or DEBONEL) [14]. Many other *Rickettsia* species have been recently identified, but are not yet well described, including the human pathogens *R. helvetica* and *R. aeschlimannii*[15,16].

Considering all the serious diseases that humans can potentially be exposed to after a tick-bite, an unambiguous diagnostic tool is essential for identifying them. The most reliable modern diagnostic tools employ serological tests, including ELISA (enzyme linked immunoabsorbent assay), Western blot, indirect immunofluorescence assay (IFA), a microagglutination test, and in the case of rickettsial infection, the Weil-Felix test [17]. Unfortunately, these methods are only indirect and do not allow illnesses to be diagnosed in the early stages of infection. Another major limitation of serology is cross-reactivity [18], application of the non-standardized antigen preparations and discrepancies in test procedures among laboratories can lead to different test results. Furthermore, identification of *Candidatus* N. mikurensis using serology is presently not possible and *A. phagocytophilum* and *E. chaffeensis* antigens do not interact with *Candidatus* N. mikurensis antibodies [19]. The primary approach for detecting *Candidatus* N. mikurensis therefore relies on PCR-based methods.

Molecular biology approaches offer the advantages of directly detecting these pathogens during early infection along with better taxonomic classification. The most common techniques employ conventional, nested, or quantitative PCR (qPCR) targeted to a genus or species specific gene, such as 16S rDNA gene (*rrs*)*, gltA*, *omp, ospA* or *ospC*[20-23]. Another method, commonly used for identifying *B. burgdorferi* s.l., targets the 5S-23S rDNA (*rrfA*-*rrlB*) intergenic spacer followed by genotyping using RFLP or SSCP [24,25]. These tests target the rDNA genes because they are minimally affected by horizontal gene transfer. Typically, these genes have hypervariable regions, specific for each bacterial genus, which are flanked by conserved regions [26].

The more recent, microarray-based techniques are high-throughput large-scale screening systems for the simultaneous identification of several target amplicons. DNA microarrays are used in many fields of research, including transcription profile analysis and DNA-DNA or protein–protein interactions. Microarrays have been developed for the identification of microorganisms in soil extracts [27], for the detection of multiple pathogens [28-30] and for differentiating between different *Borrelia* genospecies [31]. These techniques employ DNA or RNA as a template for the preparation of a target product which is suitable for passive hybridization with complementary DNA fragments or oligonucleotides bound to the surface of a slide. The stringency and hybridization efficiency is regulated by solution composition and temperature.

An alternative to the DNA microarray is an electronic microarray - biosensor, which can be prepared using standard complementary metal oxide semiconductor (CMOS) technology. This “smart” biosensor uses an electric field to regulate the stringency, transport and active hybridization of nucleic acids [32,33]. An electronic microarray based on the genus-specific variability of the *rrs* gene has already been developed for the detection of marine bacterial species [34].

In this study, we report the development of a detection system combining a second generation DNA microarray with qPCR for the detection of pathogens in vectors or in clinical samples. A second generation DNA microarray is basically an epoxy glass slide with bound capture oligonucleotides, which code for the hypervariable regions of the *rrs* gene, specific for each bacterial genus. The target DNA is amplified, Cy5-labeled using nested PCR and passively hybridized with capture probes on the microarray. We also developed qPCRs employing the genus-specific, hypervariable regions of *rrs* for *Coxiella* spp., *Francisella* spp. and *Rickettsia* spp. to confirm the DNA microarray results.

## Methods

### Bacterial isolates and genomic DNA preparation

A DNA microarray was designed to detect bacteria from *Borrelia* spp., *Anaplasma* spp., *Francisella* spp., *Rickettsia* spp., *Coxiella* spp. and *Candidatus* N. mikurensis. The DNA of *A. phagocytophilum, R. africae, R. slovaca, F. tularensis* subsp. *holarctica* and *C. burnetii* Nine Mile phase II were from laboratory stocks [28]. The DNA from different *Borrelia* species and *Candidatus* N. mikurensis was isolated from questing ticks collected in Slovakia using the Qiagen DNeasy Blood and Tissue kit (Qiagen, Hilden Germany). Positive samples were identified using previously described PCR methods and sequenced [19,25]. Borrelial DNA was also isolated from cultures kindly supplied by Dr. Ian Livey (Baxter, Orth, Austria). DNA samples from non-targeted bacteria used as negative hybridization controls [28] were also taken from laboratory stocks.

### Sequence selection of capture probes

The sequences of the DNA microarray capture probes Bv, Be, Bg1 used for the detection of *Borrelia* spp, C1 and Cv for detection of *Coxiella* spp., Av and A3 for detection of *Anaplasma* spp., F1v, F2v, Fa and F2 for detection of *Francisella* spp. and R1, Rv and Re for detection of *Rickettsia* spp. were previously published in Blaškovič and Barák [28]. New probes for detecting the DNA of *Borrelia* spp. and *Candidatus* N. mikurensis were designed (Table 1). The sequences for the new capture probes were chosen based on the hypervariable regions of the 16S rDNA genes (*rrs*). These were identified by an alignment of 16S rDNA (*rrs*) sequences from GenBank and the Ribosomal Database Project II (RDPII) [35]. The sequences of the new capture probes were tested based on melting temperature and secondary structure prediction by Integrated DNA Technologies’ OligoAnalyzer 3.1 online software [36]. The hybridization specificity of the designed probes was also analyzed using a Blast search [37].

**Table 1:** Nucleotide sequences of PCR primers and probes

**Oligonucleotide**	**Sequence (5′-3′)**	**Target bacteria**	**Source**
**DNA microarray amplification**			
16S27f	GAGAGTTTGATCCTGGCTCAG	Almost all eubacteria	Modified oligo fD1[38]
16S1495r	CTACGGCTACCTTGTTACGA	Almost all eubacteria	Modified oligo fD1[38]
**qPCR amplification**			
CbqPCR F	GGGAAACTCGGGCTAATACC	*Coxiella* spp.	This study
CbqPCR R	CACGAGGTCCGAAGATCC	*Coxiella* spp.	This study
CbqPCR P	FAM-CCCGCTTTGCTCCAAAGAGATTATG-TAMRA	*Coxiella* spp.	This study
RcqPCR F	GCTTAACCTCGGAATTGCTT	*Rickettsia* spp.	This study
RcqPCR R	CGTCAGTTGTAGCCCAGATG	*Rickettsia* spp.	This study
RcqPCR P	HEX-CCTTCGCCACCGGTGTTCCT-TAMRA	*Rickettsia* spp.	This study
FrqPCR F	ATTAAAGGTGGCCTTTGTGC	*Francisella* spp.	This study
FrqPCR F2	ATTAAAGGTGGCTTTCGGGC	*Francisella* spp.	This study
FrqPCR R	ACCAACTAGCTAATCCAACGC	*Francisella* spp.	This study
FrqPCR P	Cy5-AGGCTCATCCATCTGCGGCA-BHQ2	*Francisella* spp.	This study
**Capture probes**			
A3	CGGCTATCTGGTCCGGTACTGAC	*Anaplasma* spp.	[28]
Av	GCTGAATGTGGGGATTTTTTATCTCTGT	*Anaplasma* spp.	[28]
Be	AAGGGTGGAATCTGTTGATATCAGG	*Borrelia* spp.	[28]
Bg1	CTGGTGTAAGGGTGGAATCTGTTGA	*Borrelia* spp.	[28]
Bg2	TCAGAAAGAATACCGGAGGCGAAGG	*Borrelia* spp.	This study
Bsp1	GGAATAAGCTTTGTAGGAAATGGCAAAGTGATGACG	*Borrelia* spp.	This study
Bv	ACTTGGTGTTAACTAAAAGTTAGTACCGA	*Borrelia* spp.	[28]
Bv2	TATCAGGAAGAATACCGGAGGCGAA	*Borrelia* spp.	This study
C1	AATATCCTTGGGCGTTGACGTTACC	*Coxiella* spp.	[28]
Cv	ACTAGCTGTTGGGAAGTTCACTTCTTAGT	*Coxiella* spp.	[28]
F1v	ACTAGCTGTTGGAGTCGGTGTAAAGG	*Francisella* spp.	[28]
F2	TAGAGGAATGGGGAATTTCTGGTGT	*Francisella* spp.	[28]
F2v	ACTAGCTGTTGGATTCGGTGTAAAGG	*Francisella* spp.	[28]
Fa	AATAGCCTTGGGGGAGGACGTTAC	*Francisella* spp.	[28]
NM	CTATTTAAACTAGAGATCGAGAGAGGATAGTGG	*C.* Neoehrlichia mikurensis	This study
R1	TAGAGTRTAGTAGGGGATGATGGAA	*Rickettsia* spp.	[28]
Rv	GCTAGATATCGGAAGATTCTCTTTCGG	*Rickettsia* spp.	[28]
Re	GTGGTCGCGGATCGCAGAGA	*Rickettsia* spp.	[28]

For qPCR, three genus-specific oligonucleotides and dual-labeled probe sets were designed. The first set bound exclusively to the *Coxiella* spp. 16S rDNA gene (*rrs*), the second set was specific for the *Rickettsia* spp. 16S rDNA gene (*rrs*), and the last set was designed to bind the *Francisella* spp. 16S rDNA gene (*rrs*). The unique region of the *Coxiella* spp. 16S rDNA gene (*rrs*) was identified by aligning the 16S rDNA (*rrs*) sequences and comparing them to the previously published primers and probes for the 16S rDNA gene (*rrs*) of *Coxiella burnetii*[22]. This region was used to design oligonucleotides and dual-labeled probes using GenScript (GenScript USA Inc., Piscataway, NJ, USA). The same strategy was used to generate oligonucleotides and dual-labeled probes for the *Francisella* spp. and *Rickettsia* spp. [39,40]. Like the DNA microarray capture probes, the qPCR probes and oligonucleotides were validated based on melting temperature, predicted secondary structure folding and hybridization specificity as described above.

### PCR amplification

The sequences of all oligonucleotides and the probes used in this study are listed in Table 1.

#### PCR amplification for DNA microarray

The 16S rDNA (*rrs*) target genes of the targeted bacteria were amplified by nested PCR. The first cycle used 3 μl of genomic DNA, 1× high yield buffer complete with 2 mM MgCl_2_ (Jena Bioscience, Germany), 200 μM of each dNTP, 1 μM of 16S27f (forward) and 16S1495r (reverse) primers (Table 1) and 1U Taq Pol (Jena Bioscience, Germany). The primers 16S27f and 16S1495r are slightly modified fD1 and rP2 general eubacterial primers [38]. The second cycle was used to incorporate the fluorescent labeled Cy5-dUTP into the PCR product of the first amplification. The incorporation was performed as recommended by the manufacturer. Thus, the total 20 μl reaction volume contained 1× high yield buffer complete with 2 mM MgCl_2_ (Jena Bioscience, Germany), 100 μM of dATP, dCTP, dGTP, 50 μM of dTTP, 50 μM Cy5-dUTP (Jena Bioscience, Germany), 0.5 μM 16S27f and 16S1495r primers (Table 1) and 1U Taq Pol (Jena Bioscience, Germany). 1 μl of the PCR product from the first cycle was used as the template for the second cycle. The cycling conditions in both PCRs were the same. The initial denaturation was performed for 2 minutes at 94°C followed by 30 cycles of 94°C for 30 seconds, 52°C for 30 seconds, 72°C for 1 minute and 30 seconds. The program ended with final elongation at 72°C for 5 minutes.

#### PCR amplification for quantitative PCRs (qPCRs)

TaqMan probes for qPCRs were synthetized by Microsynth AG, Austria. The CbPr probe was covalently bound at the 5′end with a FAM fluorophore and at the 3′end with a TAMRA quencher; the RLOqPCRPr probe was covalently bound at the 5′end with a HEX fluorophore and at the 3′end with a TAMRA; and the FrqPCRPr probe was covalently bound with Cy5 at the 5′end and BHQ-2 at the 3′end. The cycling conditions for all qPCRs were the same. The initial denaturation was performed for 2 minutes at 95°C, followed by 40 cycles at 95°C for 25 seconds and 50°C for 1 minute. The reaction mixture consisted of 300 nM of forward and reverse primes, 200 nM dual-labeled probes, 1**×**TaqMan Master Mix (Bioron, Germany), 4 mM MgCl_2_ and 5 μl of template DNA.

### DNA microarray preparation and scanning

Epoxy coated slides were used for DNA microarray prefabrication. The procedure was performed as recommended by the manufacturer (Corning Incorporated, USA). Briefly, the microarray capture probes were diluted in a printing solution, consisting of 150 mM sodium phosphate, pH 8.5 and 0.01% SDS, and printed onto slides in a final spotting concentration of 30 nM. The epoxy slides were spotted at room temperature in 55–70% relative humidity and stored overnight at room temperature. All capture probes were spotted onto slides in triplicate. These prefabricated slides were blocked in a prehybridization solution (5× SSC, 0,1% SDS and 0.1 mg/ml BSA) at 42°C for 1 hour, washed 3 times in 0.1× SSC for 5 minutes and once more in purified water for 30 seconds. After washing, the slides were dried by centrifugation at 1 600 × g for 2 minutes. The Cy5-labelled target PCR products from the nested PCRs were diluted in a hybridization solution consisting of 5× SSC, 10% formamide, 0.1% SDS and 0.1 mg/ml of sonicated salmon sperm DNA, denaturated for 5 minutes in boiling water, shortly spun down, and cooled to room temperature. The target PCR products were pipetted onto microarray slides and covered with cover slips. The hybridization was performed at 42°C for 12–16 hours. After hybridization, the microarray slides were immersed in 2× SSC and 0.1% SDS at 42°C to gently release the cover slips from the slides and washed again for 5 minutes in 2× SSC and 0.1% SDS at 42°C. The final washing consisted of two washes in 1× SSC for 2 minutes at room temperature and two washes in 0.1× SSC for 1 minute at room temperature. The slides were dried by centrigufation at 1 600 × g for 2 minutes and scanned at 635 nm on a MARs Micro Array Scanner (DITABIS - Digital Biomedical Imaging Systems AG, Germany) with SpotScout Pro at 50 μm resolution. The fluorescence intensity for a given spot is represented by the mean feature pixel intensity at 635 nm minus the median background intensity at this wavelength (F635 Mean – B635). Since all slides were spotted in triplets, the reported measurements are the mean values of three measurements. An “M probe” consisting of mixed PCR fragments from the first PCRs was used as a positive control (details were previously published in Blaškovič and Barák [28]. M probe fragments were spotted onto the microarray plates in three places as capture probes. This was done in triplicate. In addition to their use as a positive control, the M probes also aided in grid location during the scanning and made it possible to performing the final negative sample evaluation. The lower border of a saturated spot was defined as 25% of the saturated spot area. All fluorescence intensity values were normalized with respect to the M probe and are reported as percentages of the M probe intensity (defined to be 100%).

### Determination of the limit of detection (LOD)

The concentration of genomic DNA isolated from the targeted tick-borne bacteria was quantified using a GeneQuant spectrophotometer (LABFISH, Germany) and the number of copies per μl was calculated using an on-line DNA copy number calculator [41]. The DNA was diluted to a starting concentration of 10^5^ copies/μl for *Coxiella* spp. and *Rickettsia* spp. and 10^4^ copies/μl for *Francisella* spp. This starting solution was then serially diluted 10-fold to prepare a series of solutions from 10^5^ or 10^4^ copies of genomic DNA (gDNA) per μl down to 1 copy/μl (that is, there were six dilutions: 10^5^, 10^4^, 10^3^, 10^2^, 10^1^, and 10^0^). To determine the microarray LOD, 1 μl of these diluted DNAs were used as templates for the amplifications of the *rrs* gene and the products of these first PCRs were Cy5-labeled using nested PCRs as described above. The labeled amplicons were then hybridized with the prefabricated microarray and scanned and evaluated.

A very similar approach was used to determine the detection limit of qPCR. The same DNA dilutions were used as the templates and genus specific oligonucleotides and probes were used to amplify the *rrs* genes.

The highest target DNA dilution which still returned a positive result was determined the detection limit of the DNA microarray or qPCR. Three independent experiments were run for each dilution series. Each qPCR experiment consisted of one of the target gDNA dilutions and a mix of non-target gDNAs as negative control. To determine the detectable copy number, an absolute quantification method was employed. The mean quantification cycle (Cq) was converted to a log starting quantity using a linear equation derived from the standard curves.

## Results

### Development of a DNA microarray for the detection of tick-borne pathogens

A DNA microarray is an efficient and simple tool for detecting a wide spectrum of tick-borne pathogens in a single step. A first generation DNA microarray for detecting tick-borne bacteria was developed previously by Blaškovič and Barák [28]. In that study, the amplification of target DNA by symmetric/asymmetric PCR appeared to be quite complicated. The authors were not able to obtain a single stranded amplicon using asymmetric PCR when DNA from *B. burgdorferi*, *C. burnetii* and *R. africae* was used as a template. They therefore recommended using symmetric PCR for the amplification of all targets involved in the study. The limit of detection (LOD) was also not determined for this assay.

The first steps for upgrading this DNA microarray were to improve the amplification of the 16S rDNA (*rrs*) gene to enable detection of all bacteria present in the analyzed samples and to increase the efficiency of Cy5-dUTP incorporation into the PCR products. A nested PCR was designed whose first cycle was used to amplify the target gene in high yield. In the second cycle, Cy5-dUTP was incorporated into the PCR amplicon. Crucial for the success of this PCR was the selection of a Taq-polymerase, which was able to both efficiently incorporate modified nucleotides into PCR fragments and to amplify the gene in high yield. Several types of Taq-polymerases, including DyNAzyme EXT DNA Polymerase, Taq DNA Polymerase, (Thermo Fisher Scientific, USA), *Taq* DNA Polymerase with Standard *Taq* (Mg-free) Buffer (New England Biolabs, USA), and many cycling conditions were tested (not shown). The best Taq-polymerase appeared to be Taq Polymerase/high yield from Jena Bioscience (Germany). The final annealing temperature was 52°C. In both nested PCR cycles, the same, so-called “catch-all” primers 16S27f and 16S1495r were used. These primers are slightly modified versions of the previously published eubacterial primers fD1 and rP2 [38]. 16S27f is the same as fD1 but with an additional G at the 5′ end. Modification of rP2 included CT addition to the 5′ end of 16S1495r and truncation of three nucleotides from the 3′ end (Table 1). The Cy5-labeled PCR products were precipitated in the presence of ammonium sulphate and ethanol [42] and the pellets were resuspended in the required volume of hybridization solution (see Methods).

### Coupling of target Cy5-labeled amplicons with capture probes

The capture probes Bv, Be, Bg1, C1, Cv, Av, A3, F1v, F2v, Fa, F2, R1, Rv and Re were previously designed to bind Cy5-labeled PCR-generated fragments encoding the 16S rDNA genes of *Borrelia* spp., *Coxiella* spp., *Anaplasma* spp., *Francisella* spp. and *Rickettsia* spp., respectively [28]. Even though the hybridization conditions and post-hybridization washes had been changed in the present study, they still reacted specifically. To increase the ability of the assay to detect the targeted bacteria, new capture probes for *Borrelia* spp. (Bg2, Bsp, Bv2) and *Candidatus* N. mikurensis (NM) were designed (see Table 1). All capture probes were printed onto epoxy slides in triplets in final concentrations of 30 μM. A Cy5-labeled PCR product encoding the 16S rDNA (*rrs*) of *Borrelia* spp., *Coxiella* spp., *Anaplasma* spp., *Rickettsia* spp. and *Francisella* spp. was generated by amplifying purified DNA from bacterial stocks. These Cy5-amplicons were then hybridized with the capture probes. Cy5-amplicons encoding the 16S rDNA of *Candidatus* N. mikurenses were generated using DNA isolated from ticks. The positivity of the tick for *Candidatus* N. mikurenses was analyzed in a previous study by qPCR [43].

The ability of the individual capture probes to bind the same target DNA differs depending on the status of the Cy5-amplicons or the quality of the spotted probes or epoxy slides. Our results clearly show that the coupling of capture probes with Cy5-PCR fragments generated from DNA isolated from ticks was not as efficient as that with Cy5-PCR amplicons generated from purified DNA. Thus, the measured fluorescence intensity of the M probe (positive control) and positive capture probes had to be normalized after scanning. The fluorescence intensity is expressed as the mean feature pixel intensity of the positive spot at 635 nm minus the median background at 635 nm (F635 Mean – B635).

**Figure 1:**
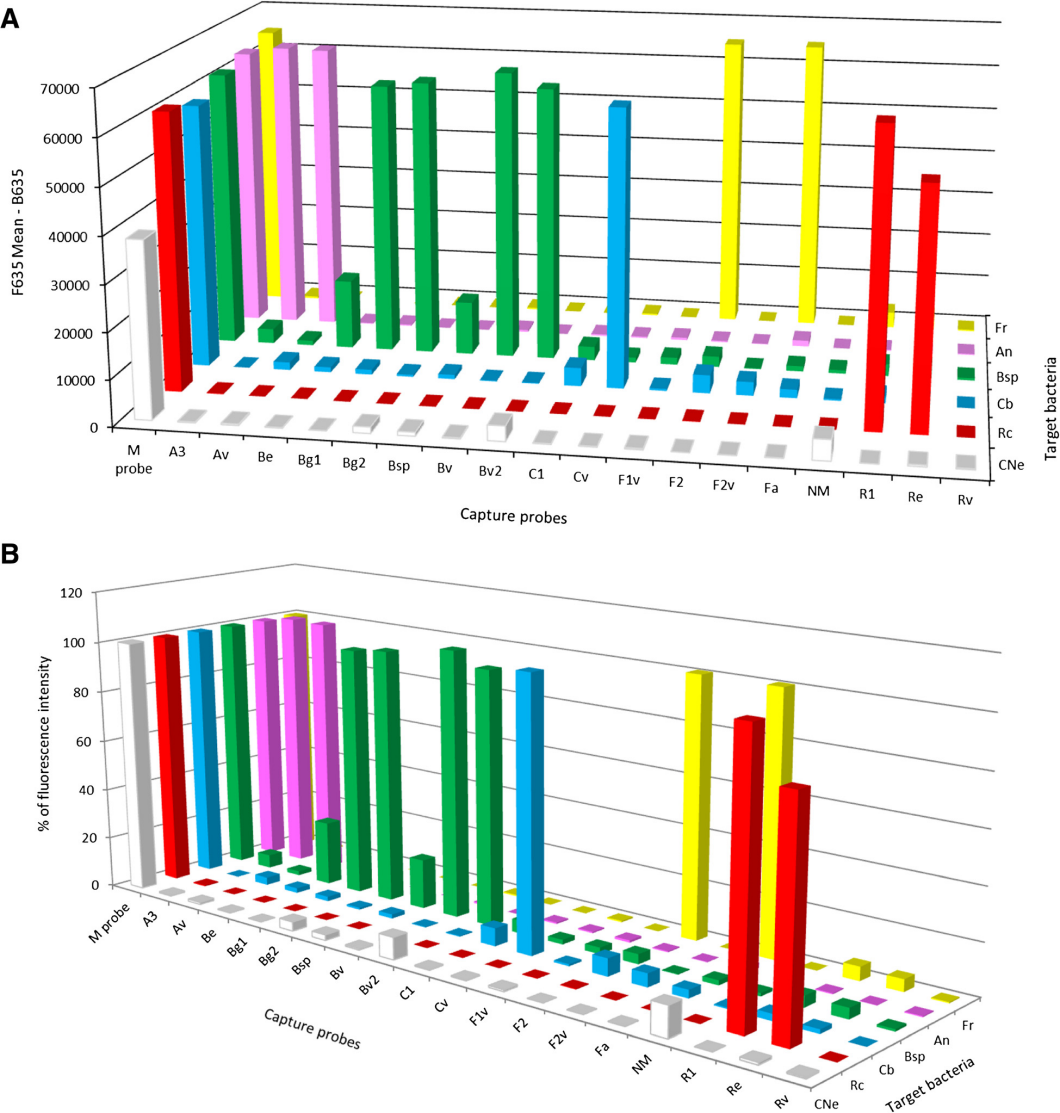
**Specificity of capture probes and target bacteria detected by the DNA microarray. (A)** Fluorescence intensity at 635 nm (F634Mean-B635) of specific capture probes coupled to target Cy5-labeled amplicons. **(B)** Fluorescence intensity expressed as a percentage of the fluorescence intensity of the capture probe in relative to 100% of the fluorescence intensity of the M probe (positive control). All capture probes are listed in Table 1. The targeted bacteria were (CNe) *Candidatus* Neoehrlichia mikurensis, (Rc) *Rickettsia* spp., (Cb) *Coxiella* spp., (Bsp) *Borrelia* spp., (An) *Anaplasma* spp., (Fr) *Francisella* spp.

The fluorescence intensity of the M probe and the positive capture probes for *Coxiella* spp., *Borrelia* spp., *Francisella* spp., *Rickettsia* spp. and *Anaplasma* spp., gave much stronger positive signals than those obtained after the hybridization of a Cy5-labeled amplicon with DNA isolated from a tick coinfected with *Borrelia* spp. and *Candidatus* N. mikurensis (Figure 1A). In order to compare these different fluorescence values, the fluorescence intensity of each positive signal was expressed as a percentage of the fluorescence of the M probe. The DNA microarray for *Candidatus* N. mikurensis revealed a possible co-infection with *Borrelia* spp. but the percentage of fluorescence intensities was quite low, only 12% and 9% respectively of the fluorescence intensity of the M probe. Such a low signal was considered only ambiguously positive and required further analysis.

**Figure 2:**
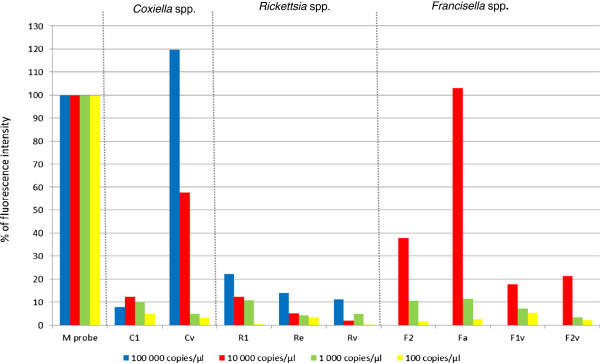
**The DNA microarray limit of detection (LOD).** LOD for *Rickettsia* spp., *Coxiella* spp., and *Francisella* spp. was determined as the highest dilution of genomic DNA that still tested as positive. 10^3^ genome copies exhibited 10% of the fluorescence intensity of the M probe, while 10^2^ genome copies produced no detectible signal; the LOD was therefore determined to be 10^3^ copies and 10% of the positive control. All capture probes are listed in Table 1. The fluorescence intensity of the capture probes is expressed as a percentage of the fluorescence intensity of the M probe.

To determine if a sample with such a low fluorescence tests positive or negative, it was necessary to determine the limit of detection (LOD) of the DNA microarray. To do this, a series of 10-fold dilutions, ranging from 10^5^ genome copies per μl down to 1 copy/μl, was prepared from DNA of the targeted tick-borne bacteria. These dilutions were then used as templates for nested PCRs and DNA microarrays. The fluorescence intensities of the capture probes were compared to that of the M probe (Figure 2). The fluorescence intensity of the Cv capture probe specific for *Coxiella* spp. was 120% when 10^5^ copies were used as template DNA, 12% when 10^4^ copies and 10% when 10^3^ copies were used. 10^2^ copies of template DNA apparently did not bind the capture probes and no fluorescence intensity could be measured; the 10 copies and 1 copy dilutions were also negative. So, the maximal dilution of template DNA, which is detectable by the DNA microarray is 10^3^ copies/μl, which gives a fluorescence intensity 10% of that of the M probe. Therefore, a sample should be evaluated as positive when the mean fluorescence intensity is at least 10% of the mean fluorescence intensity of the M Probe. Thus, samples with values close to this cut off limit will require other analyses, such as some previously published qPCR protocols [20,43], to verify their results. An analysis using the protocols of Courtney *et al.*[20] and Jahfari *et al*. [43] confirmed that the signal at the 12% level for the *Candidatus* N. mikurensis specific probe, as well as the signal at 9% for the *B*. *burgdorferi* s.l. probe both represent positive samples (data not shown).

The specificity of the DNA microarray was tested using mixed genomic DNAs from many bacterial species, including DNA from tick-borne pathogens, but excluding the target DNA for which the chip was designed. No unspecific cross-reactivity between the capture probes and genomic DNAs was detected (data not shown).

### Development of qPCR based on the variability of *rrs* specific for detection of *Francisella* spp., *Rickettsia* spp. and *Coxiella* spp

This newly developed DNA microarray represents our first line approach for the detection of tick-borne bacteria. To validate the results obtained with the DNA microarray, we used quantitative PCR. It was also necessary to verify the positive detection limits for *Candidatus* N. mikurensis (F635 Mean - B635 of 12%) and *B. burgdorferi* s.l. spp. (F635 Mean - B635 of 9%) with this method. Duplex qPCR for the simultaneous detection of *A. phagocytophilum* and *Borrelia burgdorferi* s.l. was developed by Courtney *et al*. [20]. They employed the *msp2* gene and the 23S rDNA gene, respectively, for the detection of these two pathogenic species. A real-time PCR assay for the detection of *Candidatus* N. mikurensis was developed by Jahfari *et al.*[43]. These two protocols were used to confirm the *Candidatus* N. mikurensis results from the DNA microarray. In addition, two real-time PCRs based on two putative target genes for hypothetical proteins FTT0376 and FTT0523 were developed to distinguish the pathogenic subspecies *Francisella tularensis* (subsp*. tularensis, holarctica* and *mediaasiatica*) from nonpathogenic *F. philomiragia* or *F. novicida*[44]. Because FTT0376 and FTT0523 real-time PCRs detect only the pathogenic but not the nonpathogenic *Francisella* subspecies, this approach is not suitable as a confirmatory method for our DNA microarray analysis, since all *Francisella* species is our primary target. For this reason, we developed a qPCR employing the 16S rDNA (*rrs*) gene in order to detect all *Francisella* species, and two other qPCRs, to detect the *Rickettsia* spp. and *Coxiella* spp. The primers and oligonucleotide dual-labeled probes were designed using GenScript Real-time PCR (TaqMan) Primer Design online software [45] based on the alignment of the *rrs* genes of all tick-borne bacteria of interest. These probes and primers are specific for the hypervariable region of the *rrs* gene, which are different for every genus (Table 1). Their specificities were tested against mixed genomic DNA from all possible tick-borne bacteria and other bacterial genomic DNA present in our laboratory stocks.

Because of the slight variation in the hypervariable region of the *Francisella* spp. *rrs* sequences, two forward primers were designed. Forward primer FrqPCRF preferentially binds *F. piscicida* and *F. philomiragia*, while forward primer FrqPCRF2 is specific for *F. tularensis* subsp. *holarctica, mediaasiatica, tularensis* and *novicida*. All qPCRs were specific when tested against mixed genomic DNA from other bacteria.

### Determination of qPCR detection limit

**Figure 3:**
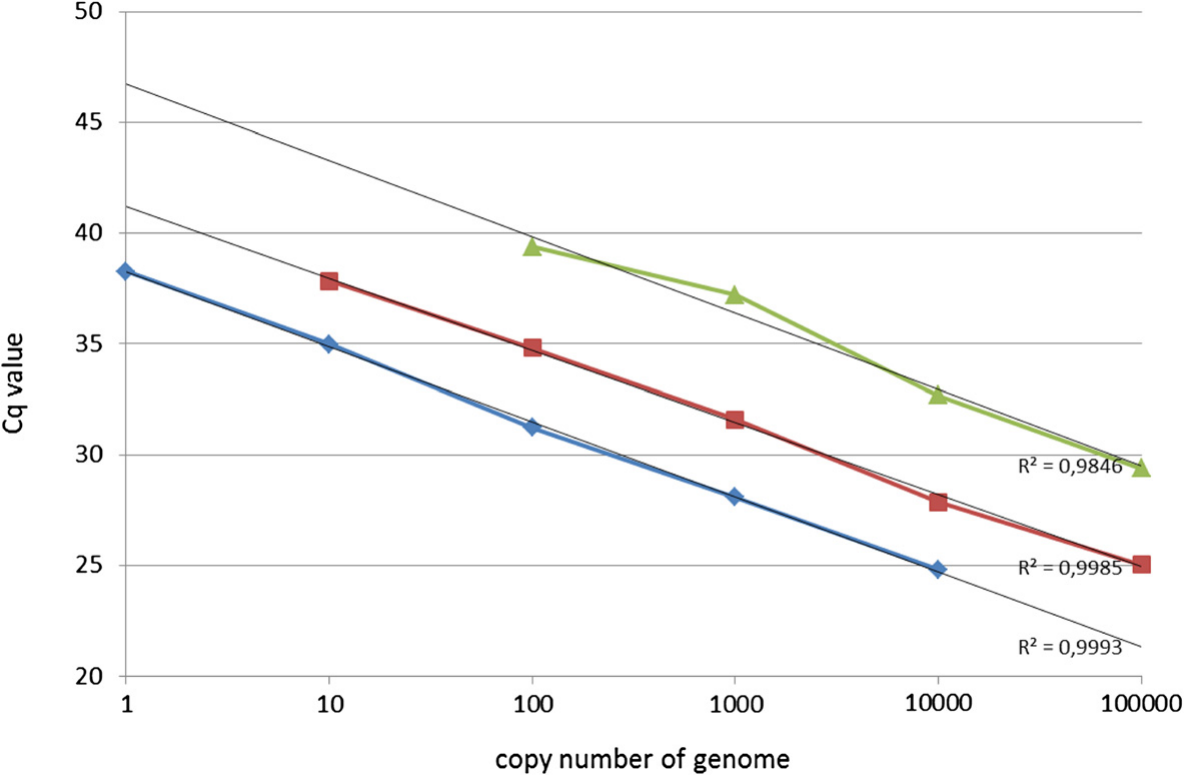
**Absolute quantification of the detectable genome copy numbers from tick-borne bacteria.** Quantitative PCRs were developed for *Francisella* spp. (blue curve), *Coxiella* spp. (red curve) and *Rickettsia* spp. (green curve). The trendlines and R^2^ values were generated using Microsoft Excel based on the average of the cycle of quantification values (Cq) and the genome copy numbers.

Limits of detection for all three qPCRs were determined based on the maximum dilution of genomic DNA from the target bacteria which still tested positive. To develop a qPCR specific for *Coxiella* spp. and *Rickettsia* spp., their genomic DNAs were diluted in a series of 10-fold dilutions, from 10^5^ copies/μl to 1 copy/μl. Due to the low concentration of genomic DNA in *Francisella* spp., the starting copy number of the series was only 10^4^ copies/μl. The absolute quantifications of the detectable genome copy number from *Francisella spp*., *Coxiella spp*. and *Rickettsia spp*. is shown in Figure 3. The standard curve amplification efficiencies (E), regression coefficients (R^2^), slopes (s) and *y*-intercept (y-int) are listed in Table 2. The amplification efficiencies of all qPCRs were 95%, 102% and 96% for *Rickettsia* spp., *Coxiella* spp. and *Francisella* spp., respectively. The sensitivity of qPCR for *Rickettsia* spp. was determined to be 10^2^ genome copies; for *Coxiella* spp., 10 genome copies and for *Francisella* spp., only 1 genome copy. Considering the mean sizes of the genomes (2.2 Mb for *Coxiella* spp., 2 Mb for *Francisella* spp. and 1.2 Mb for for *Rickettsia* spp.), the copy numbers determined for the LOD correspond to approximately 22 fg of gDNA for *Coxiella* spp., 2.1 fg gDNA for *Francisella* spp. and 140 fg gDNA for *Rickettsia* spp.

**Table 2:** The parameters of the standard curves of qPCRs

**Template gDNA**	**s**	**R**^**2**^	**E**	**Y-int**
*Rickettsia* spp.	−3,452	0,984	94,8%	46,75
*Coxiella* spp.	−3,266	0,999	102,4%	41,2
*Francisella* spp.	−3,435	0,999	95,5%	38,294

## Discussion

Broad epidemiological studies of veterinary or human importance and clinical diagnostic laboratories require the application of high-throughput, large scale assays allowing the simultaneous detection of all possible microorganisms present in a given sample. Such methods can involve broad range PCRs, multiplex quantitative PCR, molecular beacons or DNA microarrays. Usually these methods employ universal genes, such as the 16S rDNA *rrs* gene, the 23S rDNA gene, or, occasionally, genes specific for each bacterial genus or species identified in previous studies. In the last decade, DNA microarrays have become one of the most powerful approaches for the simultaneous detection of several bacterial species. They have many possible applications, including identifying bioterror agents [46] and detecting causative pathogens in clinical samples or epidemiological studies [31,47,48]. In clinical diagnostics, it is essential to know all possible co-infections of the patient in order to consider all potential complications, and thus prescribe an effective treatment. Along with other methods, DNA microarrays allow the detection of all agents of a multiple infection in one step.

The major focus of this study was to develop an easy to use, second generation low-density DNA microarray for the simultaneous detection of the many kinds of bacteria present in tick samples. This technique has potential applications in clinical and veterinary laboratories and is also suitable for broad epidemiological studies. The DNA microarray consists of genus-specific capture probes for *Borrelia* spp., *Coxiella* spp., *Anaplasma* spp., *Francisella* spp. and *Rickettsia* spp. that were designed for the first generation DNA microarray [28] along with new capture probes Bsp, Bg2 and Bv2 which were designed to increase the possibility of detecting all *Borrelia* species. In addition, a specific NM probe was designed to detect the newly emerged tick-borne bacterium *Candidatus* N. mikurensis. The modified solution compositions, hybridization conditions and post-hybridization washes did not affect specificity either of the original or the new capture probes. Capture probe specificity was analyzed by hybridization with Cy5-dUTP labeled PCR fragments generated by PCR on a template containing mixed genomic DNA from other, non-target bacteria.

The sensitivity of the first generation DNA microarray was not tested [28], and thus it was necessary to test the sensitivity of the second generation DNA microarray by determining the limit of detection (LOD). The LOD of our second generation DNA microarray was determined to be the highest dilution of the target genomic DNA that still tested as positive. The LOD was determined to be 10^3^ target genome copies based on hybridization with specific capture probes at different dilutions. Given the mean genome sizes of the targeted bacteria, the limit of detection is about ~ 1–2 pg of genomic DNA. This sensitivity is comparable to that of the low-density DNA microarrays developed to detect tick-borne bacteria such as *Borrelia* spp. [31] or other array techniques developed to detect potential biological weapons, with detection limits ~10^2^-10^4^ target genome copies [29,30].

Differences were observed in the fluorescence signal intensities produced by the DNA microarray, depending on whether the target DNA was directly purified from bacteria, or was isolated from an infected tick. These differences are likely due to the presence of junk DNA from the tick and a low concentration of the target bacterial DNA. The crucial steps of PCR amplification and Cy5-labeling on such targets are also more complicated. Both of these factors can lead to a low level of fluorescence intensity following hybridization, making interpretation of a positive signal difficult. This situation was observed when genomic DNA isolated from a tick co-infected with *Candidatus* N. mikurensis and *Borrelia* spp. was used as a template for DNA microarray analysis. The mean fluorescence of the capture probes was at the detection limit. It was therefore necessary to develop qPCRs using dual labeled TaqMan probes (Table 1). Since duplex qPCR protocols have been developed to detect *A. phagoctytophilum* and *B*. *burgdorferi* s.l. [20], and real-time PCR protocols exist for *Candidatus* N. mikurensis [43], the presence or absence of these pathogens could be verified. Quantitative PCR protocols were developed to detect *Rickettsia* spp., *Coxiella* spp. and *Francisella* spp. Oligonucleotide probes were designed according to the hypervariable regions of the *rrs* gene, which are specific for each bacterial genus; both the specificity and sensitivity of the procedure were evaluated. As for the DNA microarray, the qPCR specificity was determined by amplifying the target gene on a template containing mixed genomic, non-target DNAs other than the genomic DNA of the target bacteria. No cross-reactivity with any of these gDNAs was observed. To test the sensitivity of each qPCR, a series of 10-fold dilutions of the target genomic DNAs from *Rickettsia* spp., *Coxiella* spp. and *Francisella* spp. were employed in the amplification, with the final efficiencies of 95%, 102% and 96%, respectively. The highest dilution that was evaluated as positive was 10^2^ genome copies from *Rickettsia* spp., 10 genome copies from *Coxiella* spp. and only 1 genome copy from *Francisella* spp.; these corresponded to approximately 140 fg of *Rickettsia* spp. genomic DNA, 22 fg of *Coxiella* spp. genomic DNA and 2.1 fg of *Francisella* spp. genomic DNA. The limits of detection for all three qPCRs assays were at the breakpoint of qPCR detection and were very similar to those of multiplex qPCR for *C. burnetii* targeted to the *com1*, *icd* and *IS*1111 genes [49], real-time PCR for *F. piscicida* targeted to the *rrs* gene [39], and qPCR developed to detect a *Rickettsia*-like microorganism, which is responsible for strawberry disease in fish [40]. The qPCRs for the detection of all three pathogens appeared to be more sensitive than the DNA microarray. The qPCR for the detection of *Francisella* spp. was more sensitive than that of either *Coxiella* spp. or *Rickettsia* spp. This may be due to the existence of three copies of the *rrs* gene in the *Francisella* genomes present in the Ribosomal RNA Operon Copy Number Database [50] compared to only one copy of the *rrs* gene in *Rickettsia prowazekii*[51] and *C. burnetii*[52].

It should be noted that many ticks harbor non-pathogenic bacteria, including *Coxiella*-like [53-56], *Rickettsia*-like [57-59] and *Francisella*-like [60,61] endosymbionts, which are in a mutualistic relationship with the tick. These primary endosymbionts can provide nutrition to the host [62]. They likely evolved over a long time and they are characterized with a reduced genome [63]. However, they still retain ribosomal RNA genes which have a relatively high level of similarity to those found in pathogenic organisms. For example, the *rrs* gene of the *Amblyoma*-associated, *Coxiella*-like endosymbiont has 93% indentity to the *C. burnetii rrs*[64]. The role of the secondary endosymbionts is unknown, but they can serve as protection against other pathogens [65]. The DNA microarray developed here, together with the qPCRs, is targeted to the hypervariable regions of *rrs* genes. A positive signal generated using this approach therefore does not necessarily indicate that the host vector contains a pathogenic bacteria. A sequence analysis of the final result is needed to distinguish between endosymbiont and pathogen in these samples.

## Conclusion

We have developed a sophisticated detection system for the simultaneous detection of bacteria present in reservoir hosts, tick-vectors, and clinical specimens, based on a second generation DNA microarray employing the genus-specific, hypervariable regions of the *rrs* gene. These hypervariable sequences were used to design capture probes for the DNA microarray as well as primers and TaqMan probes for qPCRs. The qPCRs can be used to verify the positive results from the DNA microarray. Both methods display a high level of specificity and sensitivity. The limit of detection for the DNA microarray was 10^3^ genome copies. Quantitative PCRs were developed for *Rickettsia* spp, *Coxiella* spp. and *Francisella* spp. and the limits of detection were determined. Finally, previously developed and published qPCR procedures are available for the verification of presence of the other bacteria involved in this study.

## Competing interests

The authors declare that they have no competing interests.

## Authors’ contributions

The study was designed by JM and IB. The DNA samples were provided by MD. JM and MD performed laboratory experiments. JM and IB conducted genetic analysis and evaluated microarray and qPCR data. The final manuscript was written by JM, IB and MD. All authors read and approved the final version of the manuscript.
